# Calreticulin modulates the intracellular survival of mycobacteria by regulating ER-stress-mediated apoptosis

**DOI:** 10.18632/oncotarget.17419

**Published:** 2017-04-25

**Authors:** Sung Hee Jo, Ji-Ae Choi, Yun-Ji Lim, Junghwan Lee, Soo-Na Cho, Sung-Man Oh, Dam Go, Seon-Hwa Kim, Chang-Hwa Song

**Affiliations:** ^1^ Department of Medical Science, Chungnam National University, Daejeon, Republic of Korea; ^2^ Department of Microbiology, Chungnam National University, Daejeon, Republic of Korea; ^3^ Research Institute for Medical Sciences, Chungnam National University, Daejeon, Republic of Korea; ^4^ College of Medicine, Chungnam National University, Daejeon, Republic of Korea

**Keywords:** ER stress, apoptosis, mycobacteria, calreticulin, macrophages

## Abstract

Endoplasmic reticulum (ER)-stress-mediated apoptosis is a host defense mechanism against *Mycobacterium tuberculosis* (Mtb) infection. Calreticulin (CRT) is the major calcium-binding chaperone protein. Previous reports have suggested a close relationship between the cell-surface expression of CRT and apoptosis. In this study, the role of CRT during Mtb infection was examined. The results showed that Mtb infection induces CRT production by macrophages and that CRT levels are correlated with the degree of apoptotic cell death. The enhanced production of CRT was associated with the ER stress induced by Mtb infection. A significant increase in CRT translocation from the cytosol to the plasma membrane after 24 h of infection suggested the importance of CRT localization in the induction of apoptosis during Mtb infection. An investigation of the factors associated with CRT translocation and the ability of ectopically expressed CRT to induce apoptosis showed that pretreatment with a reactive oxygen species scavenger decreased Mtb-induced CRT expression, leading to the reduction of CHOP and caspase-3 activation. The intracellular survival of Mtb was significantly higher in macrophages transfected with a CRT-specific small interfering RNA than in control cells. The key role of CRT in inducing apoptosis included its interaction with CXCR1 and TNFR1 in Mtb-infected macrophages. The CRT/CXCR1/TNFR1 complex was shown to induce the extrinsic apoptotic pathway during Mtb infection. Together, these results demonstrate that CRT is critical for the intracellular survival of Mtb, via ER-stress-induced apoptosis, as well as the importance of ER stress-mediated CRT localization in the pathogenesis of tuberculosis.

## INTRODUCTION

Tuberculosis (TB) is one of the oldest persisting human diseases despite the development of a live-attenuated vaccine and several antibiotics targeting the infectious agent, *Mycobacterium tuberculosis* (Mtb). Elucidation of how Mtb escapes host innate immune responses to survive intracellularly is important to understanding the pathogenesis of TB. In recent work, we suggested that the endoplasmic reticulum (ER) stress response induced by Mtb is an important step in the development of TB [1, 2]. ER stress is a feature of several infectious diseases [2–5] as it induces apoptosis, a critical host defense mechanism against infection, including Mtb infection [2, 6, 7]. In the case of Mtb-infected macrophages, apoptosis leads to the control of mycobacterial growth; however, mycobacteria have also acquired the ability to escape the killing mechanisms of phagocytes [8, 9].

Mycobacterial infection disrupts intracellular calcium homeostasis, leading to ER-stress-mediated apoptosis via the release of Ca^2+^ [1, 10]. The calcium chaperone calreticulin (CRT) resides mainly in the ER, where it participates in protein folding, maturation, and trafficking [11]. CRT is also involved in the regulation of immune responses [12], and its exogenous addition causes profound biological effects involving diverse cellular functions [13–15].

Apoptosis is associated with plasma membrane alterations, including those resulting from the translocation of intracellular molecules such as phosphatidylserine and CRT to the cell surface, which leads to the recognition and removal of apoptotic cells [16]. Cell-surface CRT also contributes to the phagocytic uptake of cancer cells and dying cells [16, 17]. Accordingly, we hypothesized that, during Mtb infection, CRT over-expression in macrophages causes their apoptosis, thereby providing potent control of intracellular mycobacteria. Therefore, in this study, we examined the functional roles of CRT associated with ER stress during mycobacterial infection as well as the effect of CRT expression on the intracellular survival of Mtb.

## RESULTS

### CRT translocation in macrophages is associated with the mycobacteria-induced ER stress response

**Figure 1:**
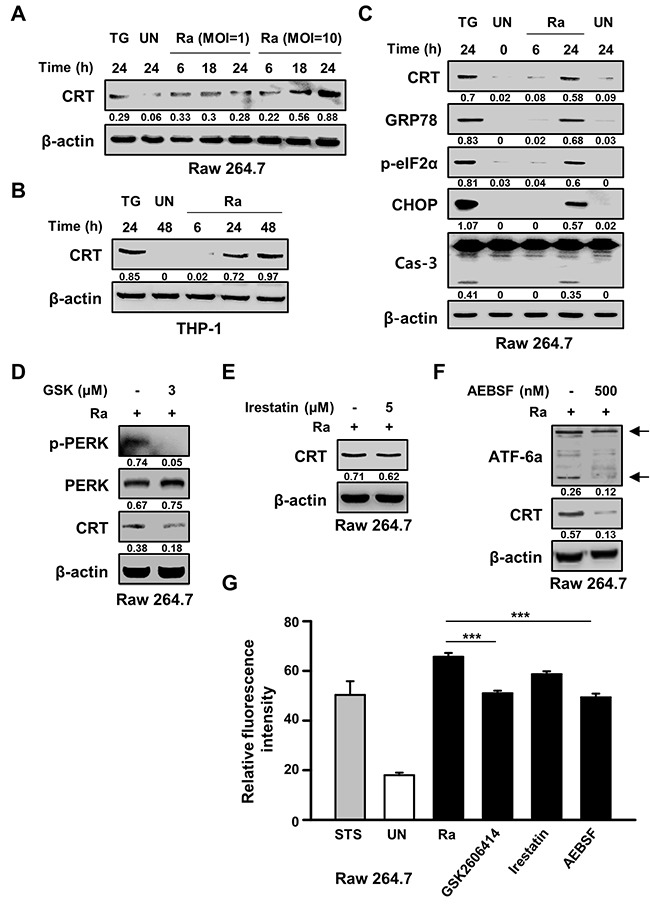
*Mycobacterium tuberculosis* (Mtb) infection induces calreticulin (CRT) production and the endoplasmic reticulum (ER) stress response in macrophages **(A)** Raw 264.7 cells were infected with Mtb H37Ra at a multiplicity of infection (MOI) of 1 or 10 and then incubated for the indicated times. Western blot analysis of CRT expression. **(B)** THP-1 cells were incubated with Mtb H37Ra (MOI=10) for the indicated times. Western blot analysis of CRT expression. **(C)** Raw 264.7 cells were infected with Mtb H37Ra (MOI=10) and then incubated for the indicated times. The levels of CRT, GRP78, p-eIF2α, CHOP, caspase-3, and β-actin determined by western blotting. **(D-G)** Raw 264.7 cells were pretreated with specific inhibitors for 1 h and then infected with Mtb H37Ra (MOI=10) for 24 h. CRT production was analyzed by western blotting in the absence or presence of **(D)** GSK2606414 (3 μM), **(E)** irestatin (5 μM), or **(F)** AEBSF (500 nM) for 24 h. **(G)** Flow cytometric analysis of the cell-surface expression of CRT in the absence or presence of ER-stress-specific inhibitors at 24 h post-infection. Western blot data presented are representative of three independent experiments. Numbers below the blot indicate the intensities ratios of each target protein to the β-actin control in each lane. Thapsigargin (TG; 500 nM) and Staurosporine (STS; 500 nM, 18 h) served as the positive control. The data are means ± SD of three independent experiments. ***p<0.001.

The ability of Mtb H37Ra to induce CRT production in macrophages was determined by examining levels of the CRT protein post-infection. After 24 h of Mtb infection at a high multiplicity of infection (MOI), CRT production was increased. This result was confirmed in the human monocyte-derived cell line THP-1, in which CRT production was significantly increased after 24 h of infection with Mtb H37Ra (Figure 1A, 1B). Since CRT is localized mainly in the ER, we investigated the relationship between CRT production and Mtb-mediated ER stress responses. The ER stress markers GRP78, p-eIF2α, and CHOP were significantly induced after 24 h of infection with Mtb strain H37Ra, coinciding with peak CRT production and significant activation of caspase-3 (Figure 1C). In Raw 264.7 macrophages pretreated with specific ER stress pathway inhibitors prior to Mtb H37Ra infection, western blots showed that CRT production induced by the bacteria was reduced by specific inhibitors of the ATF6 and PERK signaling pathways but not by an inhibitor of the IRE1 signaling pathway (Figure 1D–1F). These results were confirmed by flow cytometry, which showed a reduction in CRT production in response to specific inhibitors of the ATF6 and PERK signaling pathways (Figure 1G). These findings suggest the involvement of these pathways in the cell-surface expression of CRT following Mtb H37Ra infection.

**Figure 2:**
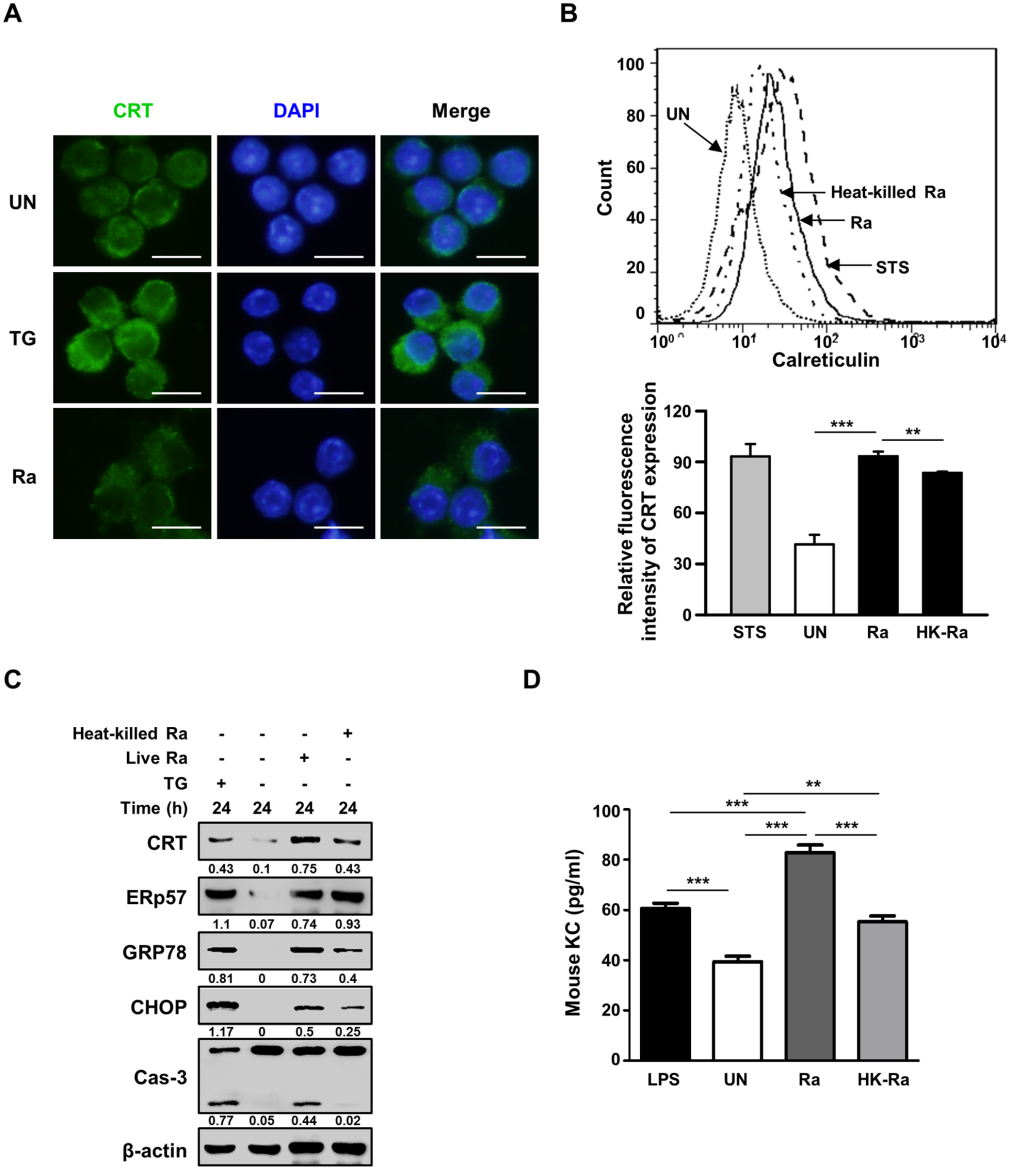
Live but not heat-killed Mtb cause translocation of CRT to the plasma membrane in macrophages **(A)** Raw 264.7 cells were infected with Mtb H37Ra (MOI=10) for 24 h. Cells were stained with anti-CRT antibody (green) for immunofluorescence. Cell nuclei were visualized by DAPI staining (blue). Scale bar: 20 μm. **(B-D)** Raw 264.7 cells were infected with live or heat-killed Mtb H37Ra (MOI=10) for 24 h. (B) Cell-surface CRT expression was analyzed by flow cytometry. Staurosporine (STS; 500 nM, 18 h) served as the positive control. **(C)** Western blot analysis of CRT, ERp57, GRP78, CHOP, caspase-3, and β-actin levels. Western blot data presented are representative of three independent experiments. **(D)** KC production was measured by ELISA at 24 h post-infection. Numbers below the blot indicate the intensities ratios of each target protein to the β-actin control in each lane. The data are means ± SD of three independent experiments. *p<0.05, **p<0.01, ***p<0.001.

Confocal microscopy of cell-surface expression of CRT after 24 h of Mtb infection showed translocation of the protein from the cytosol to the plasma membrane (Figure 2A). The requirement of live Mtb for the induction of CRT expression was investigated by flow cytometric analysis of Raw 264.7 cells after 24 h of infection with live or heat-killed Mtb H37Ra. A significant reduction in CRT expression was observed in cells infected with heat-killed but not with live Mtb (Figure 2B). The production of ERp57, which binds to CRT in the ER and co-translocates with the protein to the plasma membrane, was not affected in cells exposed to heat-killed Mtb, whereas the levels of CRT, GRP78, and CHOP were reduced. Capase-3 activation was also significantly lower in cells infected with heat-killed than with live Mtb. These data demonstrate that only the ER stress response induced by live Mtb is associated with the translocation of CRT from the ER to the plasma membrane of macrophages.

Since CXCL2 is required for CRT induction in cancer cells [18], we investigated the levels of murine chemokine CXCL2(KC) in Mtb-infected murine macrophages. The level of KC was significantly higher in murine macrophages infected with live than with heat-killed Mtb H37Ra (Figure 2D).

### Reactive oxygen species are involved in Mtb H37R-induced CRT expression in Raw 264.7 cells

**Figure 3:**
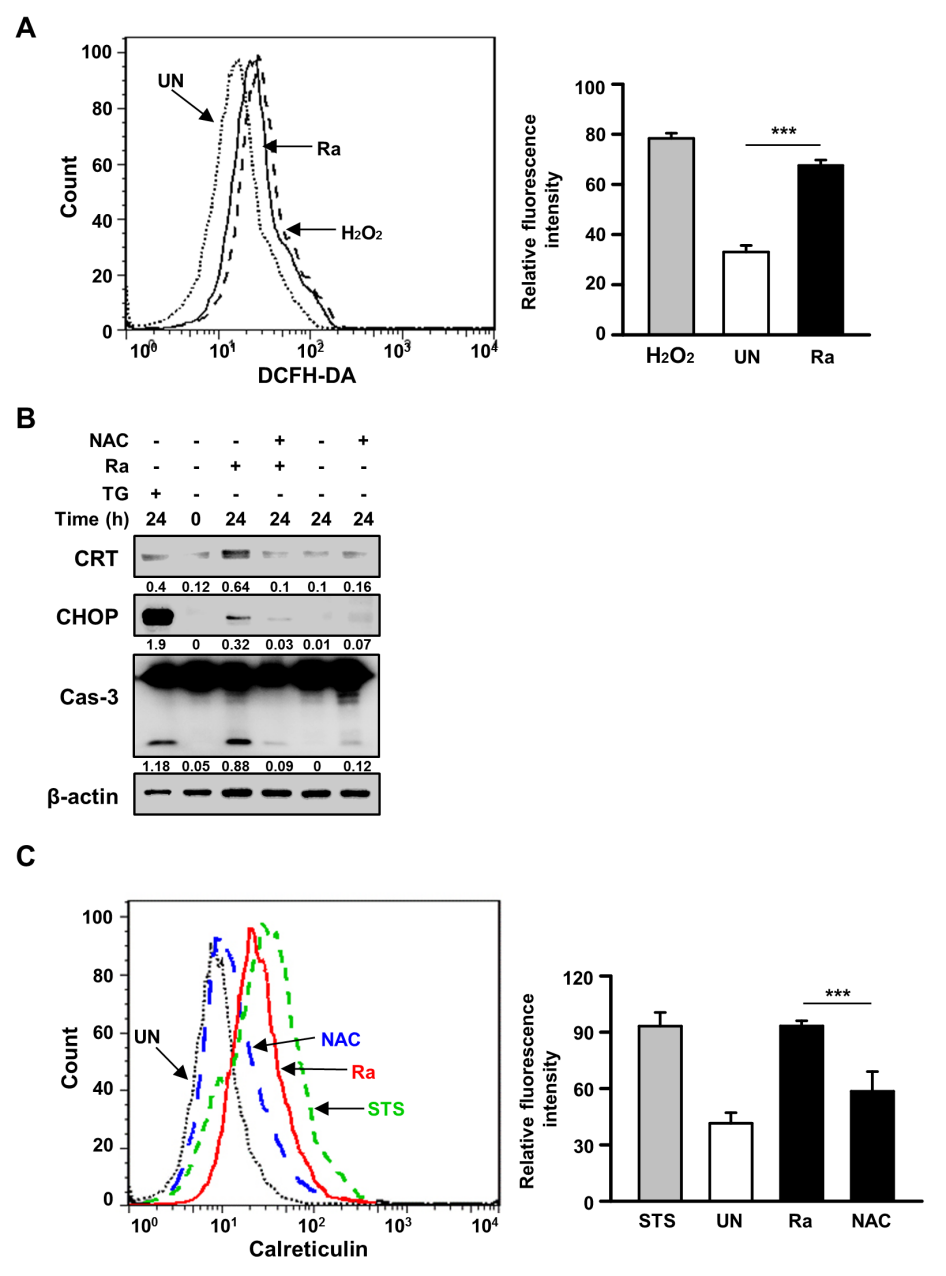
Reactive oxygen species (ROS) production is critical for the induction of CRT expression and the ER stress response in Mtb-infected macrophages **(A)** Raw 264.7 cells were stained with dichlorofluorescine diacetate (10 μM) after 24 h of Mtb H37Ra (MOI=10) infection, and intracellular ROS levels were measured by flow cytometry. H_2_O_2_ (1 mM, 1 h) served as the positive control. **(B, C)** Raw 264.7 cells were pretreated with the ROS scavenger N-acetylcysteine (NAC, 30 mM) for 1 h prior to Mtb H37Ra (MOI=10) infection for 24 h. **(B)** Cell lysates prepared after 24 h of Mtb H37Ra infection were used for western blot analysis to determine the levels of CRT, CHOP, caspase-3, and β-actin. Western blot data presented are representative of three independent experiments. **(C)** Cell-surface CRT expression was analyzed by flow cytometry using a specific antibody. Numbers below the blot indicate the intensities ratios of each target protein to the β-actin control in each lane. The data are means ± SD of three independent experiments. *p<0.05, **p<0.01, and ***p<0.001.

Because reactive oxygen species (ROS) play an important role in the induction of ER stress during mycobacterial infection [7, 10], we investigated the relationship between Mtb-mediated CRT production and ROS synthesis. ROS production was increased after 24 h of Mtb H37Ra infection (Figure 3A), but pretreatment with the ROS scavenger N-acetylcysteine (NAC) significantly reduced not only CRT levels but also CHOP levels as well as caspase-3 activation. To determine whether NAC also reduces CRT translocation from the ER to the plasma membrane, CRT expression on the plasma membrane of Raw 264.7 cells was assessed by flow cytometry. Mtb-induced CRT expression was significantly reduced by NAC pretreatment (Figure 3C). These data suggest that ROS production is critical for the induction of CRT expression on the plasma membrane of macrophages.

### The CRT/CXCR1/TNFR1 complex in macrophages is an important regulator of apoptotic cell death during mycobacterial infection

**Figure 4:**
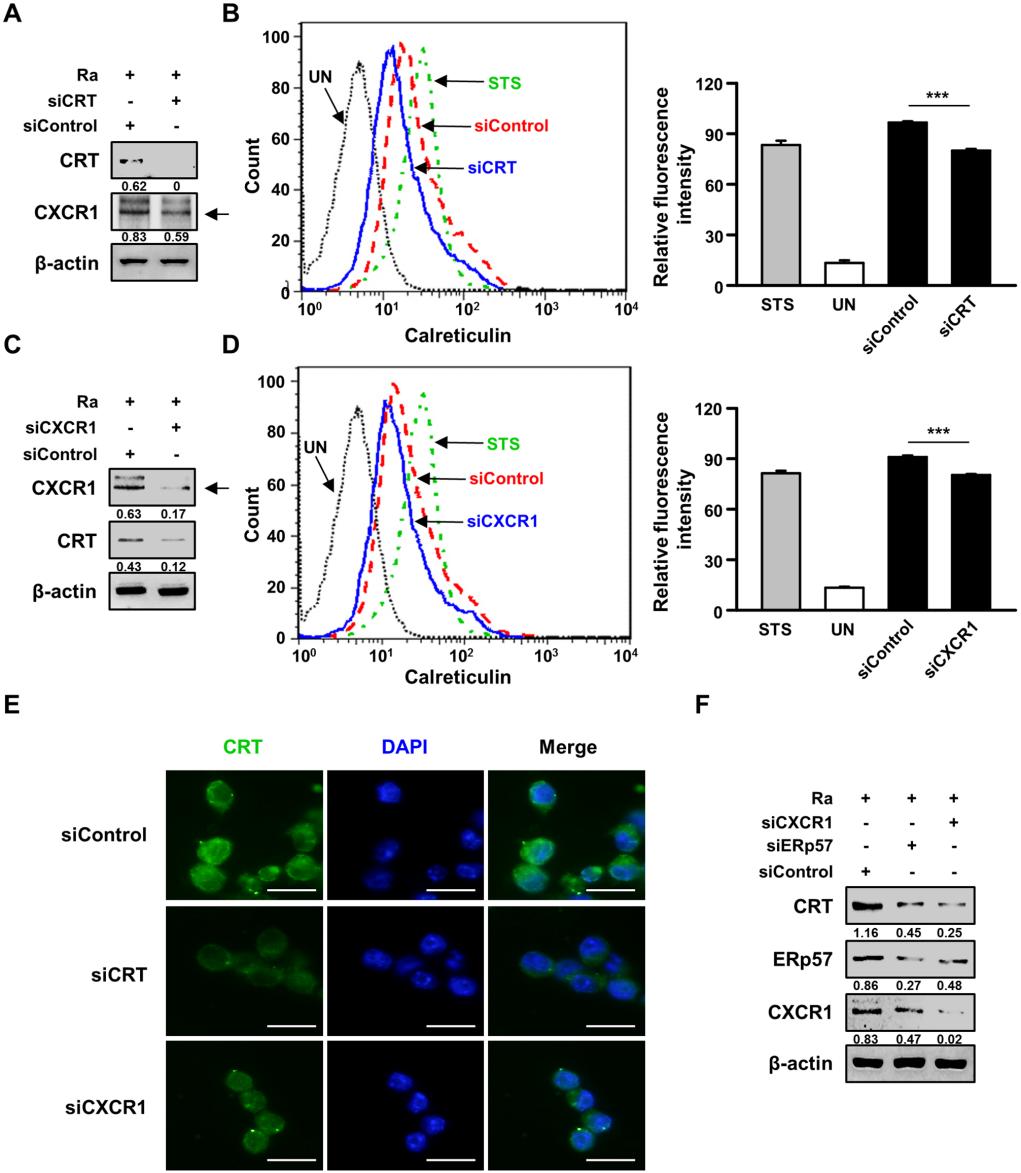
The interaction between CRT and CXCR1 activates the extrinsic apoptotic pathway during Mtb infection **(A-E)** Raw 264.7 cells were transfected with an siRNA targeting CRT (siCRT) or CXCR1 (siCXCR1) or with a non-specific siRNA (siControl) and then infected with Mtb H37Ra (MOI=10) for 24 h. **(A, C)** The production levels of CRT and CXCR1 were then analyzed by western blot analysis. **(B, D)** The cell-surface expression of CRT was analyzed by flow cytometry. Staurosporine (STS; 500 nM, 18 h) served as the positive control. **(E)** CRT staining (green) was visualized by fluorescence microscopy. Cell nuclei were visualized by DAPI staining (blue). Scale bar: 20 μm. **(F)** Raw 264.7 cells were transfected with siCXCR1 or an siRNA targeting ERp57 (siERp57) and then infected with Mtb H37Ra (MOI=10) for 24 h. The production levels of CRT, ERp57, and CXCR1 were then analyzed by western blotting. Western blot data presented are representative of three independent experiments. Numbers below the blot indicate the intensities ratios of each target protein to the β-actin control in each lane. The data are means ± SD of three independent experiments. *p<0.05, **p<0.01, and ***p<0.001.

A previous report suggested that down-regulation of CXCR1 reduces CRT exposure on the plasma membrane [18]. We therefore investigated whether the interaction of CRT with CXCR1 activates Mtb-induced apoptosis. Raw 264.7 cells were subjected to small interfering RNA (siRNA)-mediated down-regulation of CXCR1 or CRT and then infected with Mtb H37Ra for 24 h. CRT expression on the plasma membrane of the infected cells was determined subsequently (Figure 4A–4D). Membrane exposure of CRT was significantly lower in CXCR1 knockdown cells than in control cells (Figure 4C, 4D). These results were confirmed by CRT protein translocation from the cytosol to the plasma membrane in Mtb-infected cells, as revealed directly by confocal microscopy (Figure 2A). Activation of the extrinsic apoptotic pathway during Mtb infection subsequent to the interaction of CRT with CXCR1 was confirmed by confocal microscopy. The level of CRT expression was significantly reduced by siCXCR1 (Figure 4E). Since ERp57 is important for the cell-surface translocation of CRT [15], and CXCR1 is associated with the exposure of CRT on the surface of stressed and dying cells [18], we evaluated the effect of ERp57 knockdown on CXCR1 production during Mtb-induced CRT synthesis. The inhibition of CRT production by siERp57 or siCXCR1 (Figure 4F) indicated that multiple interactions of at least two different proteins, including CXCR1 and ERp57, are required for CRT translocation in Mtb-infected macrophages.

**Figure 5:**
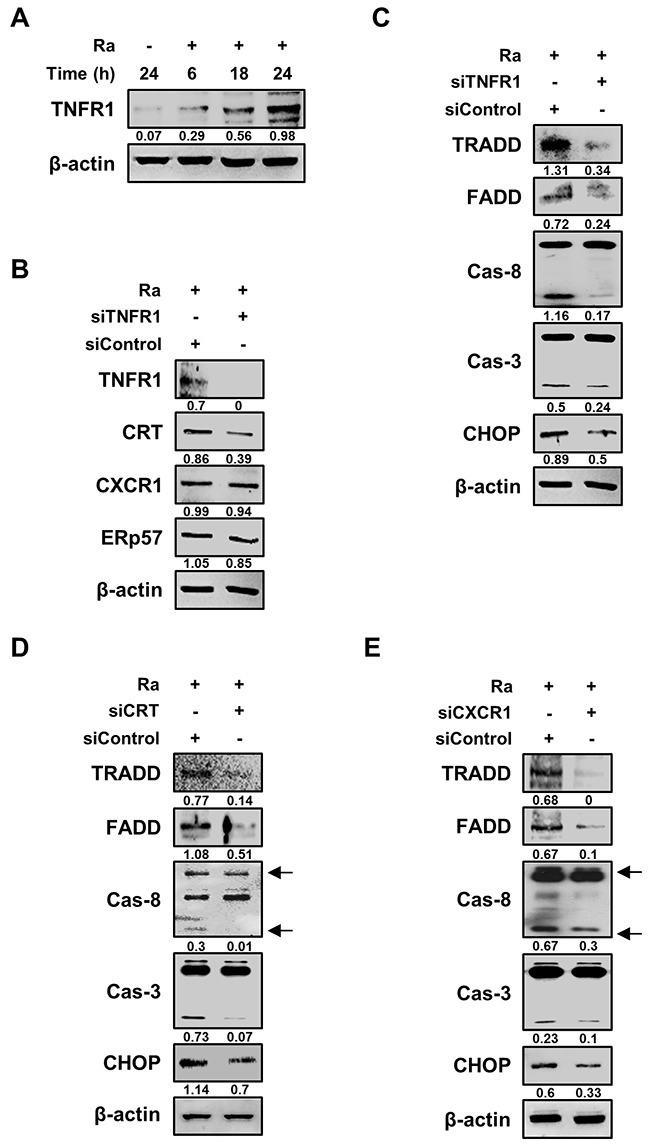
TNFR1 is essential for the activation of CRT-mediated extrinsic apoptosis during Mtb infection **(A)** Raw 264.7 cells were infected with Mtb H37Ra (MOI=10) and then incubated for the indicated times. Cell lysates were analyzed by western blotting using an anti-TNFR1 antibody. **(B)** Raw 264.7 cells were transfected with siControl or an siRNA targeting TNFR1 (siTNFR1) and then infected with Mtb H37Ra (MOI=10). The levels of TNFR1, CRT, CXCR1, and ERp57 were detected by western blot analysis after 24 h of infection. **(C–E)** Raw 264.7 cells were transfected with siTNFR1, siCRT, or siCXCR1 and then infected with Mtb H37Ra (MOI=10). Activation of the extrinsic apoptotic pathway after 24 h of infection was analyzed by western blotting using anti-TRADD, anti-FADD, anti-caspase-8, anti-caspase-3, and anti-CHOP antibodies. β-actin served as the loading control. Western blot data presented are representative of three independent experiments. Numbers below the blot indicate the intensities ratios of each target protein to the β-actin control in each lane.

We then designed a series of experiments to elucidate the relationship between CRT translocation and induction of apoptosis in Mtb-infected Raw 264.7 cells. Based on a previous report that CRT forms a complex with TNFR1 [19], we hypothesized that CRT/CXCR1 requires TNFR1, which itself forms a complex with the TNFR-associated death domain (TRADD) protein, to induce apoptosis during Mtb infection. Indeed, TNFR1 production was induced by Mtb H37Ra infection (Figure 5A), and TNFR1 knockdown reduced CRT levels, without any change in those of CXCR1 or ERp57 (Figure 5B). The levels of both TRADD and the Fas-associated death domain (FADD) protein were also reduced by siTNFR1. The death domain triggered activation of caspases, including caspase-8 and caspase-3 (Figure 5C). Under both CRT and CXCR1 knockdown conditions, the TNFR1-mediated apoptotic pathway was significantly reduced during Mtb H37Ra infection (Figure 5D, 5E). These findings indicated that the Mtb-induced CRT-CXCR1 or CRT-TNFR1 interaction is important for the induction of apoptosis in macrophages.

### The CRT/CXCR1/TNFR1 complex is critical for reducing the intracellular survival of mycobacteria

**Figure 6:**
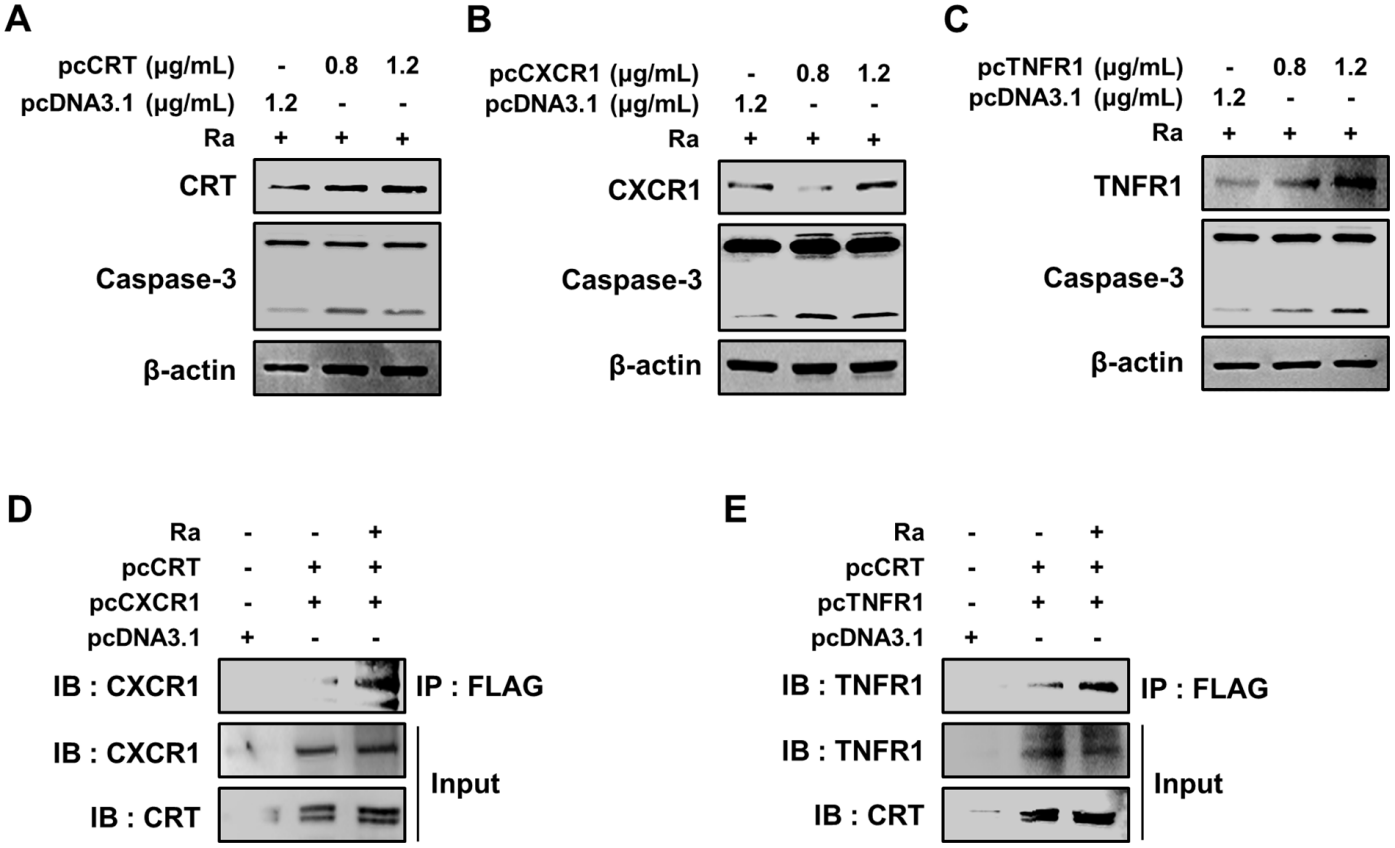
Formation of the CRT/CXCR1/TNFR1 complex increases caspase activation in Mtb-infected macrophages **(A-C)** Raw 264.7 cells were transfected with pcDNA3.1-CRT, pcDNA3.1-CXCR1, pcDNA3.1-TNFR1 or pcDNA3.1 and then infected with Mtb H37Ra (MOI=10). The overexpression of each protein after 24 h of infection was confirmed by western blotting. **(D, E)** Raw 264.7 cells were co-transfected with pcDNA3.1-CRT (1.2 μg/mL) and either pcDNA3.1-CXCR1 (1.2 μg/mL) or pcDNA3.1-TNFR1 (1.2 μg/mL) and then infected with Mtb H37Ra (MOI=10). Cell lysates prepared after 24 h of infection were immunoprecipitated using an anti-FLAG antibody (1 mg/mL) and then analyzed by western blotting using anti-CXCR1 and anti-TNFR1 antibodies. The levels of CRT, CXCR1, and TNFR1 in the total cell lysates served as the loading controls. Western blot data presented are representative of three independent experiments.

To define the molecular mechanisms that govern the induction of apoptosis, the effects of CRT, CXCR1, TNFR1, and the complex thereof in Mtb-H37Ra-infected macrophages were investigated. Each protein was overexpressed by transiently transfecting Raw 264.7 cells with pcDNA3.1(+)-CRT, pcDNA3.1(+)-CXCR1, or pcDNA3.1(+)-TNFR1. The expression of CRT, CXCR1, and TNFR1 was confirmed by western blotting, which showed their dose-dependent overexpression in Raw 264.7 cells (Figure 6A–6C). The measurement of caspase-3 levels showed that overexpression of any of the proteins induced apoptosis of Raw 264.7 cells during Mtb H37Ra infection (Figure 6A–6C). These results demonstrated the involvement of CRT, CXCR1, and TNFR1 in apoptotic cell death related to Mtb infection.

To delineate the function of the complex formed by the three proteins in mediating apoptosis during Mtb infection, the proteins were overexpressed in Mtb-H37Ra-infected Raw 264.7 cells, and the resulting complex was then immunoprecipitated using anti-Flag antibodies. Both CXCR1 and TNFR1 were detected in a complex with CRT (Figure 6D, 6E), suggesting that mycobacterial infection induces formation of the CRT/CXCR1/TNFR1 complex, which then participates in the induction of apoptosis in macrophages during mycobacterial infection.

**Figure 7:**
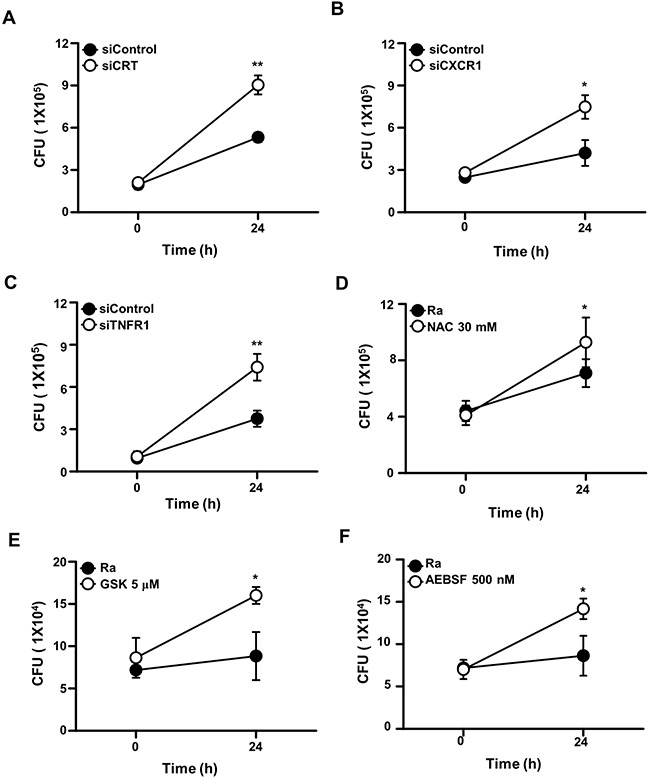
The CRT/CXCR1/TNFR1 complex regulates the intracellular survival of Mtb Raw 264.7 cells were transfected with **(A)** siCRT, **(B)** siCXCR1, or **(C)** siTNFR1 or pretreated with **(D)** NAC (30 mM), **(E)** GSK2606414 (5 μM), or **(F)** AEBSF (500 nM). After 24 h of Mtb H37Ra (MOI=10) infection, intracellular survival of the bacteria was measured by colony-forming unit (CFU) enumeration. The data are means ± SD of three independent experiments. *p<0.01, **p<0.001.

To analyze the functions of CRT, CXCR1, and TNFR1 during mycobacterial infection, the expression of each protein was inhibited using specific siRNAs in Raw 264.7 cells prior to Mtb H37Ra infection. In cells expressing CRT, CXCR1, or TNFR1 siRNA the intracellular survival of mycobacteria was enhanced (Figure 7). Similarly, pretreatment of the macrophages with the ROS scavenger NAC, a PERK inhibitor (GSK2606414), or an ATF6 inhibitor (4-(2-aminoethyl) benzenesulfonyl fluoride; AEBSF) also significantly enhanced the intracellular survival of the bacteria. These data strongly suggest that the CRT/CXCR1/TNFR1 complex mediates apoptosis through ER stress induction and thus plays a key role in suppressing the intracellular survival of mycobacteria.

## DISCUSSION

CRT is a calcium-binding chaperone involved in the immune response [17]. Its expression is induced by stress conditions such as heat shock, ER stress, and infection [20–23]. In this study, we demonstrated the antimycobacterial activity of ER-stress-mediated CRT production during Mtb infection of macrophages. In previous reports, CRT was shown to interact directly with viral proteins, thus facilitating viral infection [22, 23]. By contrast, we found that enhanced CRT production effectively reduced the intracellular survival of mycobacteria. Our results are consistent with those of studies showing the defective invasion and replication of *Legionella pneumophila* in CRT-knockout compared with control cells [24], and that CRT modulates the activity of the actin system, leading to defects in phagocytic function [25]. Whether CRT is associated with the phagocytic uptake of Mtb-infected cells is unclear, but we were able to demonstrate that the macrophage-surface exposure of CRT during Mtb infection induces apoptosis and thus reduces the intracellular survival of the bacteria. CRT relocalization to the cell surface was previously shown to trigger apoptosis in tumor cells [16]. In another study, the cell-surface exposure of CRT was shown to be dependent on interaction of the protein with ERp57 [15]. We demonstrated that only live Mtb induced the interaction of CRT with ERp57 and the subsequent translocation of both proteins to the macrophage plasma membrane. This observation implies that the cell-surface expression of CRT is part of the host defense mechanism activated by mycobacterial infection.

In addition to the folding of proteins and calcium homeostasis in the ER, CRT is involved in the regulation of cell adhesion, migration, apoptosis, phagocytosis, and immunoregulation [11, 16, 17, 25, 26]. In the present study, we demonstrated that cell-surface exposed CRT does not have direct signaling capacity but instead induces apoptosis signaling by forming a complex with CXCR1 and TNFR1. Previous reports revealed that CXCR1 knockdown reduces chemotherapy-induced CRT exposure in cancer cells, and that CXCL8 facilitates formation of the CRT/CXCR1 complex [18]. Surface CRT also complexes with TNFR1 and TRADD [19]. In an immunoprecipitation assay, we showed that CRT binds to CXCR1 and TNFR1. Studies of patients with active TB have demonstrated the importance of IL-8 as a potent inducer of T lymphocyte migration [27]. Our results underscore the contribution of the KC (homologous to human IL-8) signaling pathway in promoting CRT exposure during mycobacterial infection. In our cellular system, ER-stress-induced extracellular CRT formed plasma membrane complexes with CXCR1 and TNFR1, leading to activation of TRADD, recruitment of FADD, and ultimately caspase-8-dependent cell death. Our data suggest that mycobacterial infection induces formation of a functionally active CRT/CXCR1/TNFR1 complex that in turn plays a crucial role in the induction of apoptosis during mycobacterial infection in macrophages. Moreover, this is the first report of CRT/CXCR1/TNFR1 complex formation during mycobacterial infection.

The induction of CRT on the plasma membrane of Mtb-infected macrophages was dependent on the ATF6 or PERK pathway. In response to Mtb infection, the PERK pathway contributes to regulating the intracellular survival of mycobacteria via up-regulation of p-eIF2α [2]; its function is consistent with the antiviral role of PERK during Dengue viral infection [28]. However, activation of the PERK pathway by human cytomegalovirus infection was shown to promote infection by this virus [29]. ATF6 activation is also necessary for viral replication [30, 31]. Although *Legionella pneumophila* and *Vibrio cholera* modulate the unfolded protein response (UPR) to control host immune defenses [32, 33], the role of the UPR during bacterial infection is unclear. Its involvement in mycobacterial survival in host cells remains to be determined.

Our results also showed that mycobacteria-mediated ROS production enhanced not only the production of CRT but also its cell-surface exposure. Conversely, suppression of CRT production reduced capase-3 activation. These data suggest that Mtb induces ROS generation, and that the increased levels of ROS mediate the ER stress response, leading to CRT exposure on the macrophage plasma membrane. The subsequent formation of CRT/CXCR1/TNFR1 complexes activates caspase-8-dependent apoptosis, which results in the killing of intracellular mycobacteria. Thus, the expression of CRT on macrophage cell surfaces during Mtb infection is among the host defense mechanisms activated by mycobacterial infection.

## MATERIALS AND METHODS

### Cell cultures

Murine macrophage Raw 264.7 cells were grown in Dulbecco's minimal essential medium supplemented with 10% fetal bovine serum (FBS), penicillin (100 IU/mL) and streptomycin (100 μg/mL) at 37°C with 5% CO_2_. Cells of the human monocyte cell line THP-1 were maintained in RPMI 1640 containing 300 mg/L l-glutamine, 10% FBS, and antibiotics. THP-1 cells were incubated with 20 nM phorbol-12-myristate-13-acetate to induce their differentiation into macrophage-like cells.

### Bacteria culture and intracellular survival analysis

Mtb strain H37Ra (ATCC 25177) was obtained from the American Type Culture Collection. Mtb H37Ra was cultured in Middlebrook 7H9 liquid medium containing 10% oleic acid, albumin, dextrose, catalase, and 5% glycerol. The bacterial cells were stored at −80°C until needed. Raw 264.7 and THP-1 cells were infected with Mtb H37Ra at an MOI of 1 or 10 for 3 h. To remove extracellular bacteria, the cells were washed with phosphate-buffered saline and incubated with fresh medium without antibiotics for an additional 24 h. Mtb-infected cells were lysed in sterile distilled water and then sonicated in a water bath for 3 min to collect intracellular bacteria. The lysates were plated separately on 7H10 agar plates and incubated for 14 days. Colony counts were performed in triplicate.

### Western blot assay

Whole cells were lysed in radio-immunoprecipitation assay (RIPA) buffer (ELPIS biotech, Daejeon, Korea) in the presence of a protease inhibitor cocktail. Extracted proteins were resolved by 10% or 12% SDS-PAGE and then transferred to a polyvinylidene difluoride membrane blocked with 5% skim milk (Santa Cruz Biotechnology, Santa Cruz, CA, USA) at room temperature for 1 h. The membrane was then incubated with primary antibodies (1:1000) overnight at 4°C, washed with TBS-T, and incubated with horseradish peroxidase (HRP)-conjugated secondary antibodies (1:2000) for 2 h at room temperature. β-actin was used as the loading control. The bound antibodies were detected using a chemiluminescent HRP substrate (ECL, Millipore, Billerica, MA, USA). The blots were quantified using the Alliance Mini 4M (UVITEC).

### Quantification of extracellular KC

The concentration of KC (homologous to human IL-8) in the culture supernatants was measured using commercial ELISA kits (Mybiosource, San Diego, CA, USA), following the manufacturer's instructions. Triplicate samples were analyzed using an ELISA reader and the results compared to a standard curve.

### Antibodies and reagents

Cell lysates prepared from macrophages were subjected to western blotting. The membranes were probed with the following primary antibodies: anti-GRP78, anti-phospho-eIF2α, anti-CHOP, anti-caspase-3, anti-phospho-PERK, and anti-PERK (all from Cell Signaling Technologies, Danvers, MA, USA). Additional antibodies included anti-CRT, anti-IRE1α, anti-ATF-6α (Santa Cruz Biotechnology), anti-CXCR1 (Biorbyt, San Francisco, CA, USA), and anti-ERp57 (Abcam, Cambridge, MA, USA). The primary antibodies were used at a 1:1000 dilution. The secondary antibodies consisted of goat anti-rabbit IgG (Cell Signaling), goat anti-mouse IgG (Santa Cruz Biotechnology), and rabbit anti-goat IgG (Calbiochem, San Diego, CA, USA). β-actin was used as the loading control (Santa Cruz Biotechnology). Macrophages were pretreated with inhibitors or inducers for 1 h prior to Mtb H37Ra infection. Specific inhibitors of JNK (SP600125), ERK (PD098059), and p38 (SB203580) were purchased from Calbiochem and NAC, a specific inhibitor of ROS, from Sigma-Aldrich (MO, USA). GSK2606414 (Calbiochem), Irestatin (Axon Medchem, Groningen, Netherlands), and AEBSF (Sigma-Aldrich) were used as selective inhibitors of PERK, IRE1α, and ATF6, respectively.

### Measurement of reactive oxygen species (ROS)

Intracellular superoxide levels were measured using the dichlorofluorescine diacetate (DCFH-DA) assay. Raw 264.7 cells were infected with Mtb H37Ra for 24 h and then fixed in 4% paraformaldehyde. The fixed cells were stained with 10 μM DCFH-DA (Molecular Probes, Eugene, OR, USA) for 30 min. Positive cells were identified by flow cytometry.

### RNA interference

siRNAs targeting CRT (200 nM, Ambion, TX, USA), CXCR1 (200 nM, Qiagen, CA, USA), TNFR1 (200 nM, Bioneer Corporation, Daejeon, South Korea) and ERp57 (200 nM, Bioneer Corporation) were used to silence expression of the respective proteins. Raw264.7 cells cultured in 6-well plates were transfected with each siRNA using GenMute (SignaGen Laboratories) according to the manufacturer's protocol. After 5 h of incubation, the cells were cultured overnight in fresh medium containing 5% FBS without antibiotics and then infected with Mtb.

### Immunofluorescence

Infected cells were fixed in 4% paraformaldehyde, incubated overnight with a primary antibody targeting CRT, and then with a secondary antibody (Alexa Fluor 488 anti-goat IgG; Life Technologies, CA, USA) for 2 h at room temperature. The cell nuclei were visualized by DAPI (0.2 μg/mL) staining. The antibody- and DAPI-stained cells were identified using the Olympus DP70 fluorescence microscope (×400 magnification).

### Overexpression

CRT, CXCR1, and TNFR1 were overexpressed using vectors manufactured by Cosmo Genetech (Seoul, South Korea). Raw 264.7 cells were transiently transfected with the mouse CRT (pcDNA3.1-CRT-FLAG, 1.2 μg/mL), CXCR1 (pcDNA3.1-CXCR1-HA, 1.2 μg/mL), or TNFR1 (pcDNA3.1-TNFR1-MYC, 1.2 μg/mL) expression vector or with an empty vector (pcDNA3.1, 1.2 μg/mL) using Lipofectamine (Invitrogen, Carlsbad, CA, USA). After an overnight incubation, the transfected cells were infected with Mtb, and the intracellular survival of the bacteria was then determined. Extracts prepared from the cells were used for western blot analysis.

### Immunoprecipitation

H37Ra-infected Raw 264.7 cells were lysed in RIPA buffer. The cell extracts were incubated with an anti-FLAG antibody (1 mg/mL; Santa Cruz Biotechnology) at 4°C overnight on a rotator, mixed with protein A beads (50% slurry) at 4°C for 4 h, washed three times with lysis buffer, and then analyzed by western blotting using anti-CXCR1 and anti-TNFR1 antibodies to detect CRT binding.

### Statistical analysis

Statistically significant differences between groups were determined using the appropriate nonparametric test (Mann-Whitney or Kruskal-Wallis test). A *p-*value <0.05 was considered to indicate a significant difference and a *p*-value <0.001 a highly significant difference. Statistical analyses were performed using GraphPad Prism 5.0 (GraphPad Software, Inc., San Diego, CA, USA). The data are expressed as the mean ± standard deviation (SD). All experiments were performed at least three to five times. All experimental results were statistically evaluated using Student's t-test or one-way analysis of variance followed by Bonferroni's multiple comparison test.
